# Conjugation of HIV-1 envelope to hepatitis B surface antigen alters vaccine responses in rhesus macaques

**DOI:** 10.1038/s41541-023-00775-y

**Published:** 2023-11-24

**Authors:** Danielle Nettere, Shakthi Unnithan, Nicole Rodgers, Junsuke Nohara, Paul Cray, Madison Berry, Caroline Jones, Lawrence Armand, Shuk Hang Li, Stella J. Berendam, Genevieve G. Fouda, Derek W. Cain, Taylor N. Spence, Joshua A. Granek, Clemontina A. Davenport, Robert J. Edwards, Kevin Wiehe, Koen K. A. Van Rompay, M. Anthony Moody, Sallie R. Permar, Justin Pollara

**Affiliations:** 1 Duke University School of Medicine, Durham, NC USA; 2 Department of Molecular Genetics and Microbiology, Duke University, Durham, NC USA; 3 Department of Surgery, Duke University School of Medicine, Durham, NC USA; 4 Department of Statistics, North Carolina State University, Raleigh, NC USA; 5 Duke Human Vaccine Institute, Duke University School of Medicine, Durham, NC USA; 6 Quantitative Sciences Core, Duke University Center for AIDS Research, Duke University School of Medicine, Durham, NC USA; 7 California National Primate Research Center, University of California, Davis, CA USA; 8 Present Address: Department of Biostatistics and Bioinformatics, Duke University, Durham, NC USA; 9 Present Address: Perelman School of Medicine, University of Pennsylvania, Philadelphia, PA USA; 10 Present Address: GSK Rockville Center for Vaccines Research, Rockville, MD USA; 11 Present Address: Department of Pediatrics, Weill Cornell Medicine, New York, NY USA

**Keywords:** Conjugate vaccines, Humoral immunity

## Abstract

An effective HIV-1 vaccine remains a critical unmet need for ending the AIDS epidemic. Vaccine trials conducted to date have suggested the need to increase the durability and functionality of vaccine-elicited antibodies to improve efficacy. We hypothesized that a conjugate vaccine based on the learned response to immunization with hepatitis B virus could be utilized to expand T cell help and improve antibody production against HIV-1. To test this, we developed an innovative conjugate vaccine regimen that used a modified vaccinia virus Ankara (MVA) co-expressing HIV-1 envelope (Env) and the hepatitis B virus surface antigen (HBsAg) as a prime, followed by two Env–HBsAg conjugate protein boosts. We compared the immunogenicity of this conjugate regimen to matched HIV-1 Env-only vaccines in two groups of 5 juvenile rhesus macaques previously immunized with hepatitis B vaccines in infancy. We found expansion of both HIV-1 and HBsAg-specific circulating T follicular helper cells and elevated serum levels of CXCL13, a marker for germinal center activity, after boosting with HBsAg–Env conjugate antigens in comparison to Env alone. The conjugate vaccine elicited higher levels of antibodies binding to select HIV Env antigens, but we did not observe significant improvement in antibody functionality, durability, maturation, or B cell clonal expansion. These data suggests that conjugate vaccination can engage both HIV-1 Env and HBsAg specific T cell help and modify antibody responses at early time points, but more research is needed to understand how to leverage this strategy to improve the durability and efficacy of next-generation HIV vaccines.

## Introduction

A potent, effective, and durable HIV-1 vaccine remains an unmet critical need. The RV144 clinical trial, using a poxvirus prime and gp120 protein boost vaccine regimen, provided essential evidence that reduced risk of HIV-1 infection could be achieved with vaccination^1–3^. Subsequently, numerous trials have attempted to develop a highly protective regimen, but none have been able to improve upon the modest efficacy of RV144. The protection provided by the RV144 vaccine strategy was high at early time points, with as much as 61% reduction in infection risk at 1 year post vaccination, but it quickly waned to 31% risk reduction by 42 months^4^. Additionally, the RV144 vaccine regimen did not induce antibodies with broad and potent ability to neutralize HIV-1, which have been demonstrated to be effective at protecting from viral challenge in preclinical and clinical passive immunization studies^5–7^. Rather, in RV144, non-neutralizing antibody functions likely contributed to the reduced rate of infection observed in the vaccine arm^8^. Thus, one approach for improving upon the modest efficacy observed in RV144 is to develop a vaccine strategy that can enhance the durability of non-neutralizing responses or increase the production and breath of neutralizing antibodies. However, HIV-1 envelope protein (Env)-based immunization strategies have not yet successfully elicited antibodies with this type of durable and broad anti-viral functionality. This may be due in part to the limited ability of Env-based immunogens to recruit T cell help to drive maturation of infrequent B cell lineages that have the potential to mature into B cells that produce broad neutralizing antibodies (bNAbs). The frequency of these precursor cells may be as low as 1 in 2.4 million naïve B cells^9–11^ .

B-cell maturation and somatic hypermutation are driven by complex intracellular interactions within germinal centers. T-Follicular Helper cells (TFHs) provide essential costimulation to maturing B cells which drive these processes^12–14^. TFHs have been associated with the development of broad neutralizing antibodies (bNAbs) in natural infection and are essential for the development of long-lived affinity matured B cells in vaccination^15,16^. One approach for increasing vaccine-induced T cell help responses is to recruit pre-existing populations of T helper cells specific for other antigens with linked recognition^17^. The *Haemophilus influenzae type B* (Hib) pediatric conjugate vaccine and Pneumococcal conjugate vaccine are well-known examples of one antigen being used to provide T cell help to an unrelated antigen with limited immunogenicity^18^. In these cases, the B cell receptor (BCR) recognizes HIB or Pneumococcal polysaccharide antigen but receives help from T cells specific for the linked antigenic carrier protein. This strategy was leveraged to produce the first World Health Organization endorsed malaria vaccine, RTS,S/AS01, which uses hepatitis B surface antigen (HBsAg) as a carrier and a means to provide structural support for virus like particle formation to display the antigenic domains of the malaria circumsporozoite protein^19–21^.

Here, we evaluated the ability of a conjugate vaccine that combines HIV-1 Env and HBsAg to increase T cell help and improve vaccine elicited HIV-specific antibody responses in juvenile rhesus macaques. We chose to test this system in a non-human primate model of the pediatric population because of the close proximity to vaccination with the Hepatitis B vaccine, and because human pediatric immunization allows for completion of a long series of vaccinations prior to adolescence when the risk of HIV acquisition rises. To test our vaccine, we first immunized infant macaques according to the standard human hepatitis B virus immunization schedule to generate hepatitis B virus-specific helper T cell memory. Six months later, these animals were primed with a modified vaccinia Ankara (MVA)-based vaccine expressing either HIV-1 envelope and HBsAg or HIV-1 envelope alone and then boosted with either our Env–HBsAg protein conjugate vaccine or Env-only vaccine respectively, both administered with Alum adjuvant. We then evaluated vaccine-elicited antibody and TFH cell responses to determine if linked recognition augmented T cell help and improved antibody responses. We found that the conjugate vaccine was able to induce expansion of both HBsAg and Env specific circulating TFH cells with corresponding increase in serum CXCL13, a marker of germinal center activity. Finally, we found higher levels of binding antibodies to select Env antigens in the conjugate group. Despite these differences we found no evidence that the conjugate vaccine improved the antiviral functionality or durability of vaccine elicited antibodies. Results of this study demonstrate that conjugate vaccination can modify the vaccine-elicited response to HIV-1, but additional work is needed to improve upon this strategy.

## Results

### Study design

**Fig. 1:**
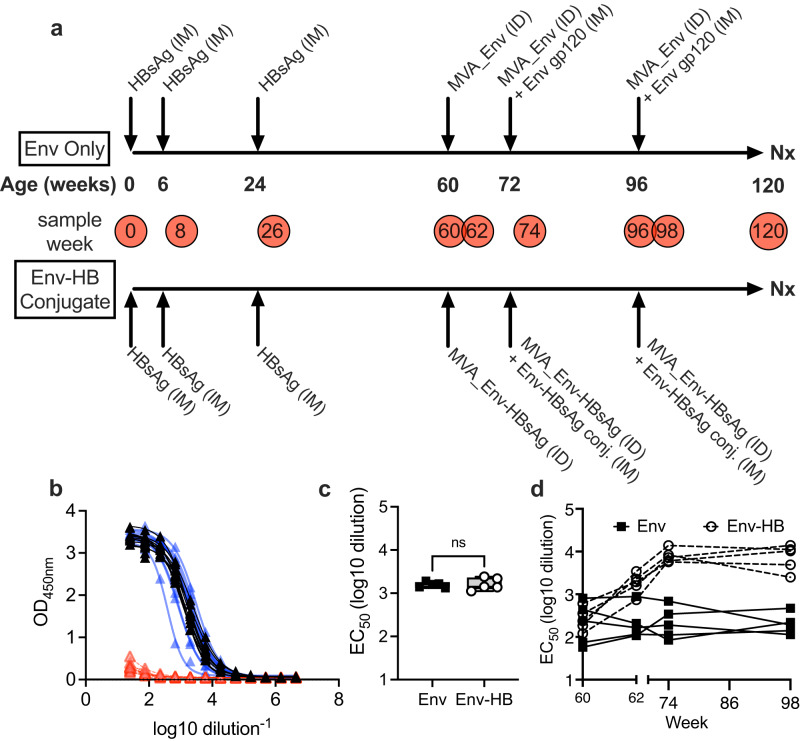
Overview of vaccination schema and induction of HBsAg specific responses. **a** HBsAg and HIV vaccination schedule and sample collection. PBMCs and serum were collected at the weeks indicated in the red circles. **b** All animals had no preexisting responses to HBsAg at time of birth (0 weeks, red lines and triangles) but all successfully responded to HB vaccination as evident by detection of HBsAg specific antibodies by ELISA 2 weeks after the second (8 weeks, blue lines and triangle) and third (26 weeks, black lines and triangles) HB vaccine immunization. **c** The titer of the HBsAg antibodies did not significantly different between vaccine groups after randomization (Mann Whitney *p* > 0.05). **d** The titer of HBsAg antibodies increased in Env-HB vaccine group but remained constant in the Env vaccine group. Box plots represent the interquartile ranges, horizontal lines indicate the medians, and error bars extend to the minimum and maximum observed values.

Ten infant Rhesus Macaques were enrolled shortly after birth and randomized to one of two vaccine arms; Env or Env-hepatitis B (HB) (Fig. 1a). Animals were enrolled in the study as births occurred and newborn macaques became available, with no ability to pre-select for animal sex. Only three females were enrolled in the study, two were assigned into the Env group, and one into the Env-HB group. All animals received the 3 dose ENGERIX-B® hepatitis B virus vaccine series, starting with the first dose given within 4 days of age, then 6 and 24 weeks of life, which mirrors the human infant vaccination schedule. All 10 of the animals produced a robust HBsAg-specific antibody response at week 26, 2 weeks after final immunization, as detected by ELISA (Fig. 1b). Importantly, we observed no differences in HBsAg antibody titers between the two groups at completion of the ENGERIX-B vaccine regimen (Fig. 1c). We next initiated a poxvirus prime and combined poxvirus and alum-adjuvanted protein boost HIV-1 vaccine regimen based on the design used in the RV144 clinical trial^1^. At 60 weeks, animals assigned to the Env-only group received an intradermal MVA-Env gp160 vaccine followed by two boosts of MVA-gp160 intradermally with Env gp120 in Alum adjuvant intramuscularly at 72 and 96 weeks (Fig. 1a). The Env-HB conjugate group monkeys received a MVA construct that co-expressed HIV-1 Env gp160 and the HBsAg as the intradermal priming immunogen, and were boosted with this MVA and a Env-HBsAg conjugate protein in Alum adjuvant via the intramuscular route at weeks 72 and 96. As expected, HBsAg antibody titers were boosted in animals that received the MVA_Env–HBsAg prime and Env–HBsAg conjugate vaccines, but not in those that received the Env-only vaccines (Fig. 1d). Both vaccine regimes were safe and well-tolerated in our cohort of infant and juvenile rhesus macaques. Peripheral blood samples were collected at 2 weeks after each vaccination and boost. (Fig. 1a).

### Env-HB conjugate vaccination expands both HB and Env specific TFH cells

**Fig. 2:**
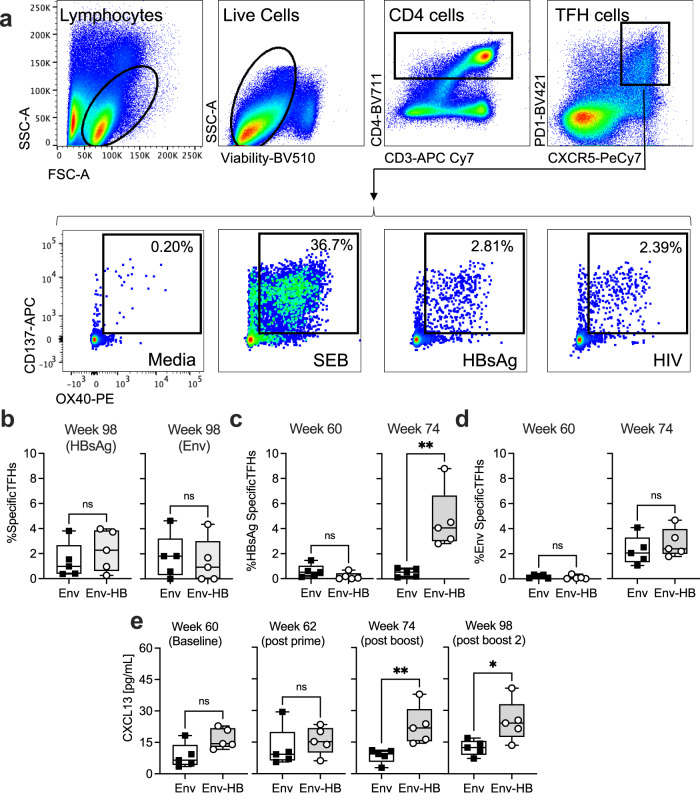
Activation induced marker assay identifies HBsAg and HIV Env specific TFH cells induced by vaccination. **a** Gating strategy used to identify antigen specific TFH cells after 18 h stimulation with SEB, or HBsAg or HIV-1 peptide and protein. **b** Frequencies of HBsAg and Env responsive TFHs out of total lymph node TFH at week 98. **c** Frequencies of HBsAg responsive TFHs out of total blood TFH at week 60 and week 74. **d** Frequencies of HIV Env responsive TFHs out of total blood TFH at week 60 and week 74. **e** CXCL13 ELISA results from serum at indicated timepoints. Comparisons were made using Mann-Whitney *U* test, *p < 0.05, **p < 0.01. Box plots represent the interquartile ranges, horizontal lines indicate the medians, and error bars extend to the minimum and maximum observed values.

We first asked if the conjugate vaccine construct could induce TFH responses specific to Env and/or HBsAg. To address this question, we used an Activation Induced Marker (AIM) assay^22^ to quantify the PD1 + CXCR5 + T-cells that respond to HB and HIV-1 Env peptides and proteins (Fig. 2a). We first evaluated the frequencies of HBsAg and Env specific TFH cells in mononuclear cells isolated from lymph nodes collected at week 98 (two weeks after final boost). As shown in Fig. 2b, the median frequency of HBsAg-specific TFH cells was modestly higher in the Env-HB vaccine group compared to the Env group, although this difference was not significant. No difference was observed between the two groups for Env-specific TFH in the lymph node. Viability of the lymph node mononuclear cells was poor (approximately 70% viable), so we therefore performed the same AIM assays using PBMCs. Within PBMCs, the PD-1 + CXCR5 + T-cells have been shown to reflect the peripherally circulating component of TFHs^15^. Peripheral blood samples from the animals at 60 weeks, 36 weeks after completion of HB vaccination, showed very low frequencies of TFH’s responding to HBsAg and none responding to Env (Fig. 2c). Two weeks after administration of the first Env-based protein boost (week 74) we observed that the frequency of HBsAg responsive TFH cells increased significantly in the Env-HB group and was unchanged in the group that received the conventional Env-only vaccine (Fig. 2c) as expected. Importantly, both vaccine arms showed an increase in the Env-specific TFH cells after the first protein boost, but the frequency of these Env-specific TFH cells did not significantly differ between the two groups (Fig. 2d). We were unable to perform AIM assays using PBMC samples collected after the second boost due to limited viable cell recovery. We therefore measured serum levels of CXCL13, a correlate of germinal center activity^23^, 2 weeks after each candidate HIV-1 vaccine dose. While there were no significant differences in CXCL13 levels 2 weeks post prime, there were significantly higher CXCL13 levels in the conjugate group after each boost, possibly reflecting the secretion from both HB and Env specific TFHs within germinal centers (Fig. 2e).

### HIV Env-specific B cells from the Env-HB conjugate vaccination group trended more towards IgG subtype and an activated memory phenotype than that of the Env-only group

**Fig. 3:**
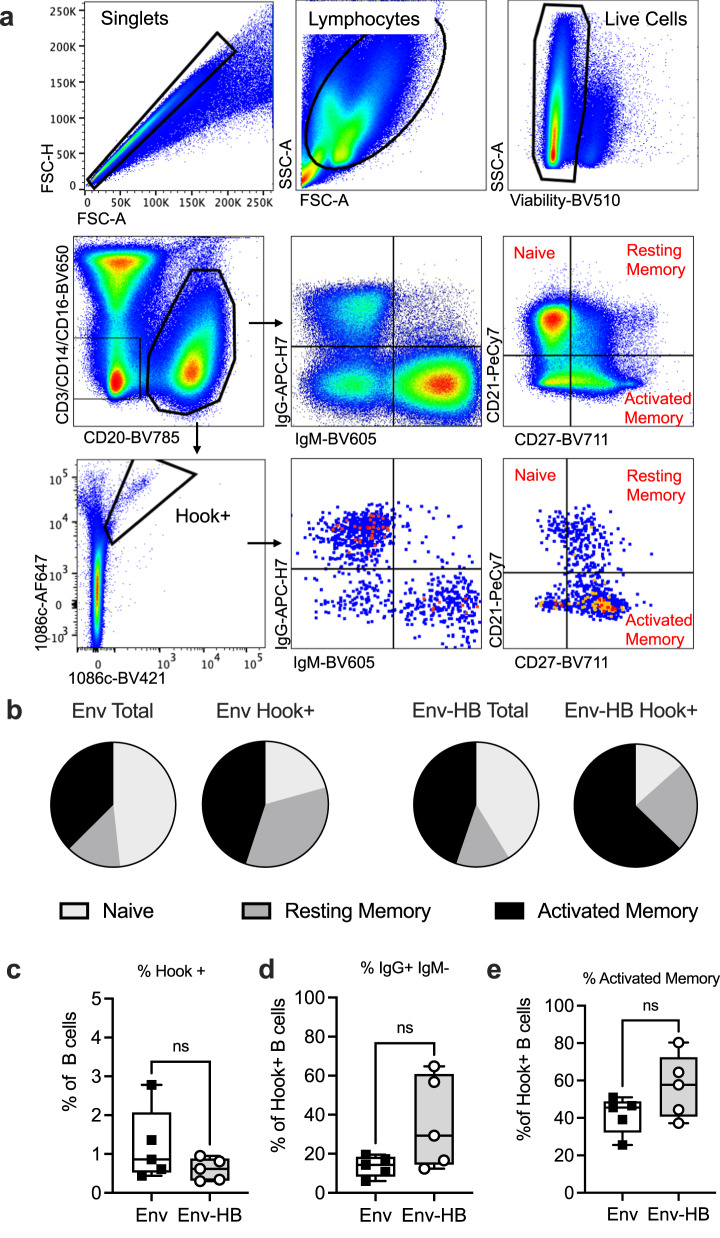
B cell antigen specific phenotyping in each vaccine arm. **a** Gating strategy used for phenotyping and sorting Env specific B cells. **b** Distribution of B cell memory subsets among total B cells and antigen specific B cell from each vaccine group. **c** Frequency of Env hook+ (Env specific) B cells among total B cells, **d** frequency of class-switched Env specific B cells that were IgG + , IgM-, and **e** frequency of Env specific B cells that were defined as activated memory cells by surface expression of CD21 and CD27. Box plots represent the interquartile ranges, horizontal lines indicate the medians, and error bars extend to the minimum and maximum observed values.

Next, we compared the phenotypes and frequencies of envelope-specific B cells from both vaccine groups. Antigen specific B cells in both vaccine groups were identified using fluorescently-conjugated C.1086 Env gp120 as hooks^24–26^. B cells were considered antigen specific if bound by both AF647 and BV421 conjugated gp120 hooks to minimize bias from non-specific binding. We assessed the frequencies and phenotypes of HIV-1 Env-specific (hook + ) B cells using flow cytometry. The gating strategy used to identify total and Env-specific B cell subsets is shown in Fig. 3a. In both vaccine groups we observed fewer naïve B-cells among the Env-specific cells when compared to total B cells, supporting the assertion that these B cells are vaccine-elicited (Fig. 3b). We also found that B cells from the Env-HB conjugate group trended towards a lower frequency of antigen specificity compared to the Env- only group (Fig. 3c), but trended towards a higher frequency of class-switched IgG+ IgM- B cells (Fig. 3d) and a higher frequency of activated memory B cells (Fig. 3e). However, none of these observations reached statistical significance.

### B cells from each vaccine arm did not vary in overall gene expression, B cell receptor (BCR) mutation frequency, BCR gene-family usage, or number of unique B cell clones

**Fig. 4:**
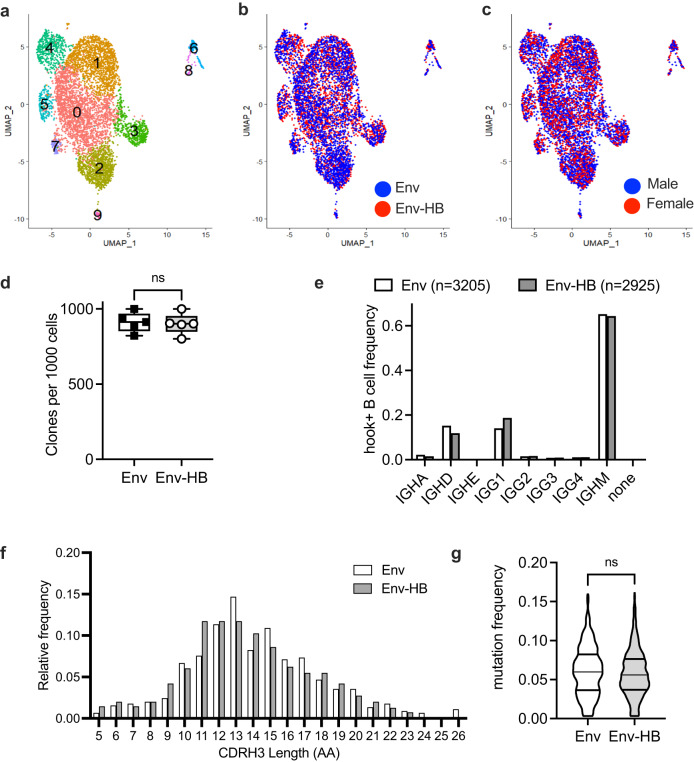
Comparisons of Env-specific B cell gene expression and BCR immunogenetics across vaccine arms. **a** Gene expression profiles of Env-specific B cells as determined using single cell RNA sequencing of Env hook+ cells identified 10 transcriptionally distinct B cell clusters. **b** Cells from Env vaccinated animals and Env-HB vaccinated animals and (**c**) from male and female animals showed overlapping distribution in each of these clusters. **d** The number of unique B cell clones per 1000 Env hook + B cells did not differ (Mann Whitney *p* > 0.05) when comparing the Env and Env–HB vaccine groups. **e** Heavy chain sequences were used to determine the BCR isotypes and subclasses, and a significant but modest increase amount of IgG1 BCR, and decrease in IgD BCR, among animals in the Env–HB group compared to those in the Env-only group was observed (Mann Whitney *p* < 0.01). Among the antigen specific B cells with IgG1 BCR, we saw a similar distribution of (**f**) CDRH3 and (**g**) no difference (Mann Whitney *p* > 0.05) in the IgG heavy chain mutation frequency when comparing the two vaccine regimens. Box plots represent the interquartile ranges, horizontal lines indicate the medians, and error bars extend to the minimum and maximum observed values.

To investigate whether conjugate vaccination modified the gene expression profiles or increased the somatic hypermutation of the Env specific B cells, Env-specific B cells were sorted by fluorescence-activated cell sorting, followed by single cell library preparation using the 10X Genomics Platform, and RNA sequencing. Two libraries were prepared for each sample, a 5’ gene expression library, and a B cell V(D)J sequencing library to simultaneously resolve the transcriptome and immunogenetic diversity of Env-specific BCRs. Our final data set included a total of 6130 cells hook+ cells, with near equal representation from the two different vaccine groups (3205 cells from Env immunized, and 2925 cells from Env–HB immunized macaques). We applied Uniform Manifold Approximation and Projection (UMAP) and graph-based clustering to reduce the dimensionality of the data and to identify distinct transcriptomic clusters. As shown in Fig. 4a, a total of 10 distinct cell clusters (clusters 0 through 9) were identified. Cells from Env vaccinated animals and Env–HB vaccinated animals showed overlapping distribution in each of these clusters, indicating overall similar transcriptomes for these two groups (Fig. 4b). Additionally, cell distributions were similar when comparing male and female rhesus (Fig. 4c), suggesting sex also did not have a discernable impact on the transcriptomes of the antigen-specific B cells we collected. We next compared the immunogenetics of antigen-specific B cells using single cell BCR sequencing. We first determined the number of unique B cell clones within the total population of antigen-specific B cells. As shown in Fig. 4d, we found no difference in the number of unique clones per 1000 Env hook+ B cells when comparing the Env and Env–HB vaccine groups, suggesting both vaccines had similar capacity to engage and expand antigen-specific B cell clonal lineages. We then compared the heavy chain sequences to determine the isotypes and subclasses of Env-specific B cells and found a significant but modest increase amount of IgG1 BCR, and decrease in IgD BCR, among animals in the Env–HB group compared to those in the Env-only group. This is consistent with the trends for higher frequencies of IgG+ and activated memory B cells observed with B cell immunophenotyping by flow cytometry (Fig. 3). Among the antigen specific B cells with IgG1 BCR, we saw a similar distribution of CDRH3 length (Fig. 4f), and no significant difference in the IgG heavy chain mutation frequency, indicating an overall similar extent of B cell maturation when comparing the two vaccine regimens.

### Antibodies induced by Env-HB vaccination both qualitatively and quantitatively differed from those in the Env- only group

**Fig. 5:**
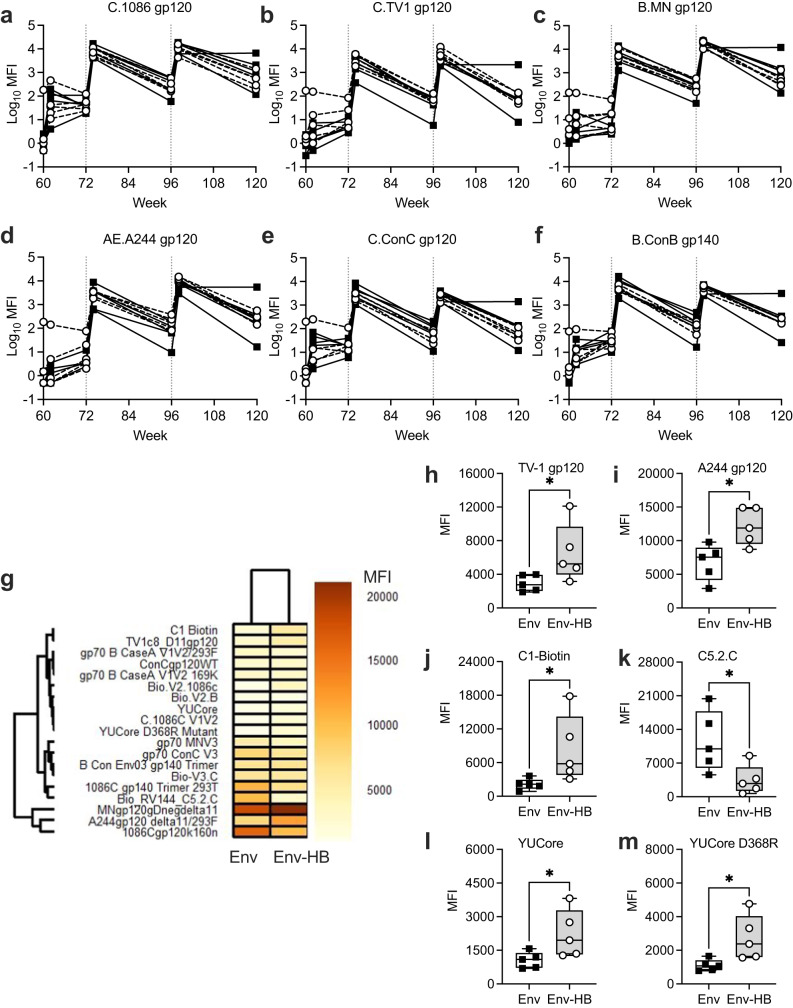
Evaluation of plasma antibody binding magnitude to envelope antigens. **a**–**f** Binding Antibody Multiplex Assay measured antibody binding to 6 unique gp120’s at each collection point. **g** Binding data for week 98 serum samples against all BAMA antigens as measured by MFI of each vaccine group and represented as a heat map. Week 98 BAMA MFI data were significantly different (Mann Whitney, *p* < 0.05) between the Env and Env-HB vaccine groups for vaccine antigens (**h**) TV-1 and (**i**) A244 gp120, (**j**) C1-Biotin, (**k**) C5.2C, (**l**) YUCore, (**m**) YUCore D368R. When controlling for false discovery rate (FDR) the adjusted *p* values for each of these observations were *p* = 0.100. Box plots represent the interquartile ranges, horizontal lines indicate the medians, and error bars extend to the minimum and maximum observed values.

We next compared the plasma antibody responses elicited by the Env-only and HB–Env conjugate vaccine. We used Binding Antibody Multiplex Assay (BAMA) to define the magnitude and specificity of envelope specific antibodies longitudinally across the study. Figure 5a–f shows levels of vaccine-elicited multi-subtype gp120/140-binding plasma antibodies over time in the Env (filled symbols, solid lines) and HB–Env (open symbols, dashed lines) vaccinated animals. We tested binding to all gp120s present in the MVA-vaccine (5A–D) and consensus B gp120 and C gp140s (5E and 5F respectively). Despite only moderate generation of binding antibodies following the prime, all the animals robustly responded to each boost and binding antibodies remained detectable at time of necropsy, 24 weeks after the final immunization. Summary data for BAMA are represented by heat map in Fig. 5g. We next compared the median fluorescent intensities (MFI) for each BAMA antigen between the two groups 2 weeks after the final immunization (week 98). We used Mann-Whitney tests to compare the BAMA response across the two vaccine groups and found that the Env-HBsAg regimen elicited higher median levels of binding antibodies to vaccine antigens TV-1 and A244 gp120 (*p* = 0.032 and 0.016, respectively), and higher levels of C1-specific (*p* = 0.016), C5.2C-specific (*p* = 0.032), and YUCore and YUCore D368R (*p* = 0.032) targeting antibodies (Fig. 5h–m). When controlling for false discovery rate (FDR) the adjusted *p*-values for each of these observations were *p* = 0.100.

### Functional responses of Env specific antibodies did not vary significantly between groups

**Fig. 6:**
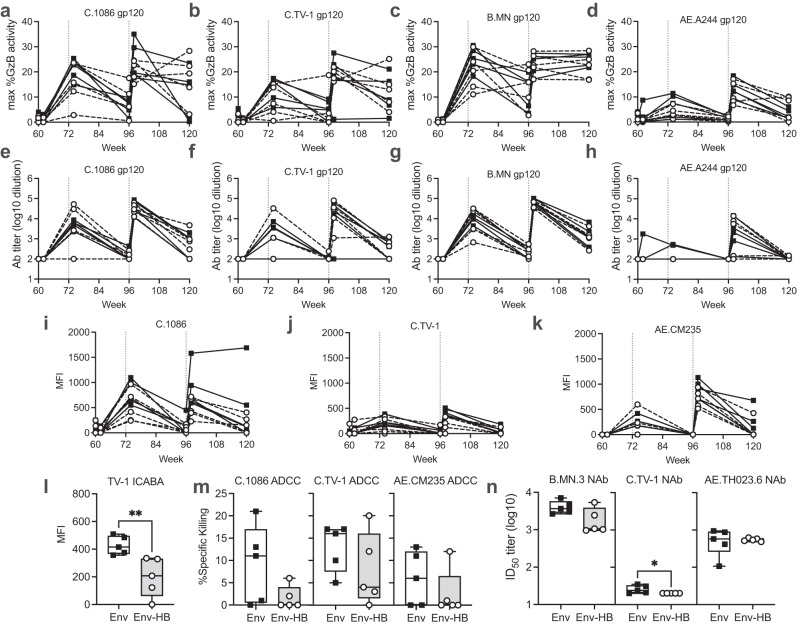
Comparison of functional antibody responses between vaccine arms. **a**–**d** Max ADCC activity as measured by Granzyme B delivery to gp120 coated target cells for 4 different gp120s over the course of the study. **e**–**h** ADCC antibody endpoint titer for assays performed with 4 different gp120s over the course of the study. **i**–**k** Binding magnitude to HIV-infected cells over time. **l** Binding to TV-1 infected cells at week 98. **m** Max ADCC activity as measured by elimination of HIV infected cells at week 98. **n** Neutralization titer (ID_50_) of antibodies at week 98. Box plots represent the interquartile ranges, horizontal lines indicate the medians, and error bars extend to the minimum and maximum observed values.

Having identified modest differences in the binding antibody response we next asked if the vaccine-elicited antibodies differed functionally in their ability to neutralize HIV or recruit effector cells for Antibody Dependent Cellular Cytotoxicity (ADCC). We first measured the ability of the antibodies in the plasma samples to engage natural killer (NK) cells to direct killing to gp120 coated target cells. We assessed ADCC activity by measuring granzyme B delivery into target cells^27^ at 6 dilutions of plasma and reported the peak granzyme B delivery (6A–D) and the ADCC antibody endpoint titer (6E–H) using 4 different gp120s. While all animals had detectable responses against each of the tested Env isolates, there was no difference in the maximum observed granzyme B activity or ADCC antibody titer when comparing the two vaccine groups. Notably, these functional responses were detected at most time points and peaked following boost immunizations, but waned at further time points after immunization. Importantly we did not observe differences in the durability of ADCC antibody responses between the two vaccine groups. We next measured the ability of the antibodies to recognize cells infected with HIV-1 using our infected cell antibody binding assay (ICABA) (Fig. 6i–k). When comparing the two groups the only statistically significant difference noted was a decreased median ability of the antibodies from the Env–HB group to recognize TV-1 infected cells at week 98 (Fig. 6l, Mann-Whitney *p* = 0.008, adjusted *p* = 0.047). These results contrast with what was observed in the BAMA assay, suggesting that the epitopes available on the surface of infected cells differ from those recognized by gp120 in the context of the BAMA assay (Fig. 5h). With regards to the ability of the serum to engage NK cells to direct elimination of the HIV infected cells, the groups were not significantly different (Mann-Whitney all *p* > 0.05) but trended towards higher activity in the Env-only group (Fig. 6m). Neutralization was also superior in the Env-only group for TV-1 but not significantly different for MN or TH023.6 (Fig. 6n). Overall, these data suggest that despite the higher overall binding to gp120s observed in the Env-HB group, the Env-only group produced antibodies with modestly better antiviral functionality.

### Identification of immune features that differentiated the Env-only and HB-Env conjugate vaccine-elicited immune responses

**Fig. 7:**
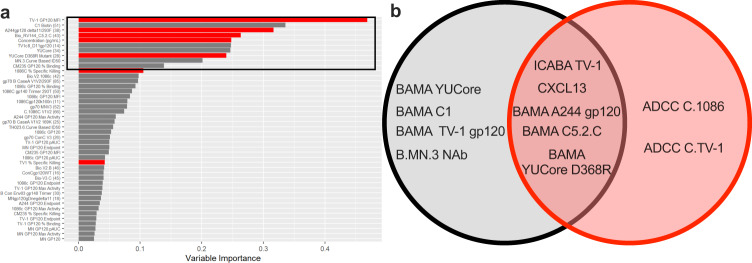
Identification of immune features that differentiated the Env-only and Env-HB conjugate vaccine-elicited immune responses. **a** A random forest model was used to identify immune features differentiating Env and Env-HB vaccinated macaques. The bar graph represents ranking of the measured immune variables that contributed to accuracy of the model (Variable importance) for the combined data set. The black box encompasses the 10 immune variables that contributed most to differences between vaccine groups. Bars highlighted in red were also identified as important (non-zero coefficient parameters) using Lasso-regularized logistic regression modeling. **b** Venn diagram representing the top 10 immunological variables that define each vaccine group by Random Forrest and Lasso-regularized logistic regression modeling.

We next sought to identify the immune features of those measured that differentiated the two vaccine groups. Due to the small sample size (*n* = 5 animals per group), and large number of immune parameters measured, a model that is robust to overfitting was needed. We therefore used a random forest model as our primary analysis approach. In addition, we also used Lasso-regularized logistic regression models as they are a common approach for classification and have been used in other HIV-1 vaccine preclinical studies^28–31^. Random forest and Lasso models were created from the combined data set for the peak immunogenicity timepoint (week 98)^28–31^. Using these models we identified the importance of immune variables from each data set along with the accuracy of the models. As shown in Fig. 7a, we found alignment between the results of random forest models and the lasso models. Among the top 10 immune variables contributing to differences between the two groups identified by random forest (grey bars), antibody binding to certain antigens and CXCL13 levels were also identified as differentiating the two groups vaccine groups using lasso (red bars). A Venn Diagram displaying the key parameters selected by each model is shown in Fig. 7b. These analyses identify immune variables that were modified by the conjugate vaccine groups, and therefore indicate variables modified by the conjugate vaccine approach.

## Discussion

HIV-1 Env based vaccine strategies trialed to date have demonstrated limited ability to induce antibodies with broad, potent, and durable antiviral activity. Part of this challenge is due to the high degree of somatic hypermutation required of B cells to develop antibodies with broadly neutralizing activity^32,33^. In natural infection it takes years of ongoing infection and strong T follicular helper responses to induce the type of broadly neutralizing antibodies that have been demonstrated to prevent infection in passive immunization studies^6,7,34,35^. The RV144 trial showed that protection could also be achieved without the induction of these responses, but only moderate levels of protection were achieved, and protection waned quickly^1^. New strategies aimed at increased antibody functionality and persistence will be essential to move the field forward towards an effective HIV-1 vaccine.

Our aim with this conjugate vaccine was to induce a more robust envelope specific TFH response to support B cell maturation and somatic hypermutation. TFH cells are involved in the formation of germinal centers, promotion of affinity maturation, selection of long-lived B cells^12–14^ and have been associated with development of dominant bNAb responses^15,16,36,37^. Thus, we hypothesized that inducing a strong TFH cell response would likely improve the quality and durability of HIV-1 vaccine elicited antibody responses. Many of the proposed Env-based immunogens are not likely to provide the ideal antigenic stimulus for TFH cells as vaccine responses are limited to T cells specific for Env^38^. This limitation is likely more severe for rationally designed, germline-targeting, minimal Env antigens such as glyco-peptides and Env-based nanoparticles which provide even fewer potential T cell epitopes^39–41^. Contrastingly, lineage and Env-swarm-based vaccine strategies likely limit TFH recall responses due to genetic diversity of the different env sequences^37,40^. Here we used the juvenile rhesus macaque model to evaluate conjugating HIV Env to HBsAg as a strategy for recruitment of additional TFH cells to provide help to Env-specific B cells and promote a higher degree of affinity maturation or drive higher titers of antibody responses. This expands upon previous work by other groups demonstrating that leveraging T cell help from non-Env based immunogens, either Gag^42,43^ or Tetanus toxoid^44^ can generate superior immune responses with VLP Env-immunogens leveraging intrastructural help in mouse models.

We tested our vaccine regimen using neonatal/young macaques as a model for human infants and children because most immunizations against preventable human diseases are administered during infancy and childhood. The high global adherence to recommended early-life immunization visits and the low overall risk of infection during childhood, especially following weaning, provides an ideal opportunity to initiate a HIV-vaccine regimen that can be completed prior to adolescence when transmission risk dramatically rises^45^. Additionally, hepatitis B virus immunization is the first vaccine received by newborns, and the regimen is completed by 6 months of age. Neonatal vaccination against hepatitis B virus elicits a durable and protective humoral immunity that typically persists for at least 15 years^46,47^. This suggests that a strong and effective TFH response is established in immunized infants by this vaccine.

We developed an innovative HIV Env–HBsAg conjugate vaccine and tested it in the juvenile rhesus macaque model to see if linked recognition could improve T cell help and drive longer lasting and higher quality HIV Env-specific antibody responses. This type of linked recognition has been proven effective in other vaccine constructs, and the proposed mechanism behind the improved responses is through leveraging existing TFH responses from the carrier antigen to driven B cell responses to the antigen of interest^17^. This strategy has been used for decades in the pediatric Hib and Pneumococcus vaccines^48^, as well as recently in the malaria vaccine^19–21^. While the successes of these platforms suggest this is a viable method for improving immune responses, the details of the immune responses elicited by conjugate vaccination has not been robustly compared to vaccination with unlinked antigen. Here we have comprehensively analyzed how antigen linking HIV-1 Env to HBsAg alters both cellular and humoral immune responses compared to unlinked antigen. We showed that the conjugate vaccine induced TFH activation of both HBsAg and HIV Env-specific T cells using the AIM assay. Additionally, compared to vaccination without conjugation, the linked recognition approach induced higher levels of binding antibodies to immunogen gp120s, as well as higher correlates of germinal center activity and trends towards more activated B cells. We think this is most likely due to activation of both HBsAg and HIV Env specific T cells. Interestingly however, these observations of enhanced activation and antibody production did not result in functionally superior antibodies in terms of neutralization activity, Fc-mediated functions, or indicators of B cell somatic mutation such as heavy chain mutation frequency and number of unique B cell clones elicited. Part of this may be due to the low number of animals enrolled in the study, which limited our statistical power. It is also possible that while we see evidence of germinal center activation with the AIM assays and CXCL13 secretion, this might have been from driving additional HBsAg-specific antibody production (Fig. 1d) which could have occurred at the expense of the HIV-1 Env-specific responses. In future studies, we will test the hypothesis that we could better leverage the envelope-specific responses to our conjugate immunogens by removing HBsAg from the MVA prime, so that the priming antigen does not expand HB-specific B cells that would over compete with the Env-specific B cells for the help provided by the conjugate protein boost. We also found that our conjugate vaccine had the ability to aggregate into larger particles, and we observed reduced antigenicity of conformational V1/V2 epitopes when the conjugate was evaluated by ELISA with well-characterized HIV-1 monoclonal antibodies (Supplementary Material). Thus, it is possible that the conformation of the conjugate protein, either the intended monomeric conjugates or the spontaneously formed aggregates, reduced presentation and availability of V1/V2 epitopes and perhaps others in vivo and contributed to the differences in binding and functional antibody responses we observed in this study. Future work will be needed to determine if antibody functionality and durability could be improved by intentionally optimizing our conjugate immunogen to promote generation of well-formed virus-like particles that present all classes of neutralizing epitopes as has recently been described for a novel SARS-CoV-2 spike-HBsAg nanoparticle vaccine^49^.

The lack of functional improvement we observed in this study may also be a consequence of the limitations inherent to gp120 immunogens. The gp120 proteins used in the vaccine were selected to match those used in recent poxvirus prime, protein boost, human clinical trials^1,50^. However, monomeric gp120 proteins are not effective for eliciting B cell responses against vulnerable neutralizing epitopes. It therefore remains possible that the conjugate vaccine strategy would have more benefit if used with vaccines based around germline targeting or native conformation antigens^40,51,52^.

A limitation of the study is the lack of ability to assess responses within the lymph nodes after each immunization. The lymph nodes collected from these animals had relatively poor viability and were limited in cell number from all but the final biopsy. We therefore also assessed antigen-specific PD1 + CXCR5+ circulating CD4 + T cells since they have been reported to reflect the circulating TFH component^15^. Additionally, due to the small animal size and blood draw limitations, only one cellular analysis could be performed at each time point, which unfortunately meant that the PBMC T cell AIM assays were performed after the first protein boost while the B cell analysis was performed after the second.

Despite the lack of a robust antibody response induced by this vaccine strategy, there is evidence that linked recognition altered how the immune system mounted a response to the Env antigens. This robust comparison of conjugate vaccination compared to Env antigen only vaccination provides insight into the specific elements of the immune response that are engaged through the addition of the HBsAg. This provides hope that this methodology could potentially be added into the toolbox of vaccine strategies to enhance the immunogenicity of HIV-1 antigens.

## Methods

### Animals and sample collection

10 Infant Indian rhesus macaques (Macaca mulatta), born from the breeding colony of the California National Primate Research Center (CNPRC, Davis), were enrolled in the study at birth and were maintained for the study duration of 120 weeks at CNPRC. The animals were negative for simian immunodeficiency virus, type D retrovirus, and simian T-cell lymphotropic virus type 1. The study was approved by the Institutional Animal Care and Use Committee of the University of California, Davis, and the CNPRC is accredited by the Association for Assessment and Accreditation of Laboratory Animal Care International (AAALAC). Animals were nursery reared for the first four months and then were moved into regular non-infectious housing for the remainder of the study. All procedures of animal husbandry and sample collections were performed according to established Standard Operating Procedures that were approved by the Institutional Animal Care and Use Committee of the University of California, Davis, and that were in compliance with the 2011 *Guide for the Care and Use of Laboratory Animals*^53^. All experimental manipulations were performed under ketamine anesthesia (10 mg/kg body weight) administered by the intramuscular (IM) route. Blood samples were collected via peripheral venipuncture at weeks 0, 8, 26, 48, 62, 74, 98, and at necropsy (week 120). Both plasma samples and Peripheral Blood Mononuclear Cells (PBMCs) were isolated from the blood draws, processed, and cryopreserved. Lymph node biopsies were collected at weeks 26, 62, 98, and 120 from a peripheral lymph node under general sedation, followed by topical anesthesia and 3 days of analgesia (ketoprofen, 2–5 mg/kg once daily). Animals were euthanized at the end of the study with ketamine sedation, followed by an overdose of sodium pentobarbital (120 mg/kg IV); consistent with American Veterinary Medical Association recommendations^54^.

### Vaccines and vaccine regimens

All 10 macaques received the ENGERIX-B® (GlaxoSmithKline, Philadelphia, PA) hepatitis B virus vaccine dose series (10 μg IM) at 0, 6, and 24 weeks post birth, which mirrors the human infant vaccine schedule. The macaques were then randomized into two groups of 5 animals, balanced for sex (4 males group A, 3 males group B). One group received the HIV Envelope (Env)-only vaccine regimen, and the other group received the conjugate HIV Env HBsAg vaccine regimen. The overall study scheme is shown in Fig. 1a.

*HIV-1 Env-only vaccine group*: At 60 weeks of age, animals in the HIV-1 Env-only vaccine group were primed with an intradermal (ID) administration of 1 × 10^7^ PFU of the MVA-1086C Env gp160 virus (MVA_Env vaccine). This conventional MVA-based vaccine was generated using red-fluorescent protein (RFP) selection and previously described methods^55,56^. Briefly, the codon-optimized, subtype C isolate 1086 Env gp160 gene (Gene bank: FJ444399.1) and the RFP reporter gene (DsRed, Clontech Laboratories, Mountain View, CA) were inserted into MVA A681 virus^56^ within an intergenic region between essential viral genes 0I8R and G1L, under the control of a synthetic early/late poxvirus promoter^55^. The RFP reporter gene used to aid in plaque selection was flanked by wild-type *lox* sequences and was removed from the final virus by Cre-mediated deletion as previously described^56^, producing MVA-1086C Env gp160 virus. At 72 and 96 weeks of age animals received booster immunizations of the MVA_Env vaccine (1 × 10^7^ PFU, ID) and alum-adjuvanted gp120 Env gp120 proteins representing subtype C isolate TV1 (GenBank EU855132.1), subtype AE isolate A244^57^, and subtype B isolate MN (GenBank AF075722.1). These envelopes were selected due to their recent use in poxvirus prime, protein boost, clinical trials^1,50^ The final Env protein immunogen was prepared with equimolar amounts of each gp120, in 2% aluminum hydroxide wet gel suspension adjuvant (Alhydrogel®, InvivoGen, San Diego, CA), administered at a final dose of 50 μg, IM.

*Conjugate vaccine group*: At 60 weeks of age, animals in the conjugate vaccine group were primed with an intradermal (ID) administration of 1 × 10^7^ PFU modified vaccinia Ankara (MVA) virus engineered to co-express HIV-1 Env protein and HBsAg (MVA_Env–HBsAg). The MVA-based vaccine was generated starting from the MVA-1086C Env gp160 virus described above. To incorporate HBsAg, the gene for HBsAg subtype adw2 WHO International Standard^58^ (Gene bank KY003230.1) was inserted into the deletion III region of the plaque-purified MVA-1086 Env gp160 virus. HBsAg was expressed under the control of the enhanced H5 promoter^59^. PCR of plaque purified virus was used to identify virus expressing both the Env and HBsAg genes, and Env and HBsAg protein production was confirmed by immunoblot (Supplementary Fig. 1A) of MVA_Env–HBsAg-infected cells.

At 72 and 96 weeks of age animals received booster immunizations of the MVA_Env–HBsAg vaccine (1 × 10^7^ PFU, ID) and an alum-adjuvanted Env–HBsAg protein conjugate (50 μg, IM). For generation of the protein conjugate vaccine, Env gp120 protein amine groups were covalently linked to HBsAg protein (subtype adw, Novus Biologicals, Centennial CO) reduced sulfhydryls with a 7.3 Å spacer using Sulfo-GMBS (ThermoFisher, Waltham, MA) according to manufacturer’s instructions. Conjugates were purified by high performance liquid chromatography (HPLC), and quality control validation of conjugation was performed using antibody-coated bead pulldown assays. Negative stain electron microscopy (NSEM) (Supplementary Fig. 1B) provided evidence that the majority of the conjugate protein was present in small, likely monomeric forms, while a minor fraction aggregated into larger particles that did not appear to form well-defined virus-like nanoparticles^60,61^. Conjugate vaccines were formed using the same 3 different Env gp120 proteins used in the Env-only vaccine: subtype C isolate TV1, subtype AE isolate A244 and subtype B isolate MN. The antigenicity of the conjugate vaccine was compared to that of the Env protein only vaccine using ELISA performed with well-characterized HIV-1 specific antibodies targeting the CD4 binding site, mannose-dependent 2G12 epitope, C1-C2 cluster A region, V1/V2 region, and V3 region (Supplementary Fig. 1C). For the final immunogen, the three different Env–HBsAg conjugates were mixed in equimolar ratios in 2% aluminum hydroxide wet gel suspension adjuvant (Alhydrogel®), at a final IM dose of 50 μg total protein.

*anti-HBsAg ELISA:* Custom ELISA using a commercially available ELISA buffer kit (ThermoFisher) and recombinant HBsAg (Abcam, Cambridge, UK) was performed to measure plasma levels of HBsAg-specific antibodies. High binding 96 well plates were coated overnight with HBsAg protein at 2 μg/mL and 4 °C in the kit’s coating buffer A (pH 7.4). The next morning the plates were washed with Wash Buffer and then blocked for 1 h in Assay Buffer. Plates were then washed, and plasma samples were added after serial dilution in Assay Buffer (1:25 starting dilution, 12 serial 3-fold dilutions for samples collected at weeks 0, 8, and 26, and 1:100 starting dilution, 6 serial 5-fold dilutions for samples collected at 60, 62, 74, and 98 weeks). Plates were incubated with plasma samples at room temperature for 1 h and then washed with Wash Buffer. Plates were then incubated with the secondary detection antibody (mouse anti-monkey IgG HRP Southern Biotech, 1:10,000 dilution in Assay Buffer) for 1 h at room temperature, washed twice with the Wash Buffer, and developed for 15 min using the kit-provided chromogen solution and stopped with an equal volume of the provided Stopping Buffer. Plates were read on a Perkin-Elmer VICTOR® Nivo^TM^ reader at 450 nm within 30 min. EC_50_ values (inverse dilutions) were calculated using non-linear curve fitting in GraphPad Prism Software (Version 10.0).

### TFH activation-induced markers (AIM) assay

TFH AIM assays were performed as described^62^. In brief, week 60 and week 74 PBMCs or week 98 lymph node mononuclear cells were thawed in R10 (10% FBS in RPMI with L-glutamine, Pen-strep and gentamycin), viable cells were counted, and then incubated in AIM media (10% Human AB serum in AIM-V serum free media) for three hours at 37 °C. Cells were then recounted, and plated in a 96 well v-bottom plate at 4 million viable cells per mL using the following conditions: Unstimulated: AIM media with 0.1% DMSO; Staphylococcal enterotoxin B (SEB) stimulated: AIM media with 0.5 μg/mL SEB; HBV stimulated: AIM media with 5 μg/mL HBsAg protein (Abcam, Cambridge, UK) + 0.5 μg/mL HBV large envelope protein peptide mix (JPT, Berline, DE); and HIV Env stimulated: AIM media with 5 μg/mL 1086cK160Nd11 and 0.5 μg/mL HIV Env Potential T cell Epitopes peptide pool (NIH AIDS Reagent program, Bethesda, MD). Cells were incubated 18 h at 37 °C and then washed with PBS 1% BSA and incubated for 30 mins room temperature with commercially available antibodies to cell-surface proteins as follows: PD-1 BV421 (Biolegend cat. 329920, clone EH12.2H7, volume 5 μL per 100), CD8a BV570 (Biolegend cat. 301038, clone RPA-T8 volume 0.62 μL per 100), CCR7 BV605 (BD Biosciences cat 563711, clone 3D12 volume 1.25 μL per 100), CD25 BV650 (Biolegend cat 302634, clone BC96 volume 1.25 μL per 100), CD4 BV711 (Biolegend 317440, clone OKT4 volume 1.25 μL per 100), CD45RA BV785 (BD Biosciences cat 741010, clone 5H9 volume 0.62 μL per 100), ICOS BB515 (BD Biosciences cat 565880, clone C398.4A volume 0.31 μL per 100), CCR6 BB700 (BD Biosciences cat 566477, clone 11A9 volume 1.25 μL per 100), OX40 PE (BD Biosciences cat 340420, clone L106 volume 5 μL per 100), CXCR3 PE-CF594 (BD Biosciences cat 562451, clone 1C6 volume 2.5 μL per 100), CD69 PE-Cy5 (Biolegend cat 310908, clone FN50 volume 5 μL 100), CXCR5 Biotin (eBioscience cat 13–9185–82, San Diego, CA, clone MU5UBEE volume 0.62 μL per 100), CD137 AF647 (Biolegend cat 309824, clone 4B4-1 volume 1.25 μL per 100), CD3 APC-Cy7 (BD Biosciences cat 557757, clone SP34-2 volume 1.25 μL per 100). Cells were then washed twice with PBS and stained with a viability dye (LIVE/DEAD Fixable Aqua Dead Cell Stain 1:800 dilution, Invitrogen cat L34957) and PeCy7-Streptavidin (Biolegend 1:800 dilution) for 20 min at room temperature. Cells were then washed with 1% PBS in BSA, resuspended in 1% PFA in PBS and acquired immediately on a LSR II Fortessa and analyzed using FlowJo Version 10.8.0 (BD Biosciences). Gates were drawn using concatenated sample files that included 5000 events from each individual sample. HBsAg and Env specific TFH frequencies were reported as the percentage of TFH cells expressing OX40 and CD137 after background subtraction of the unstimulated condition from the HBsAg and Env conditions.

### CXCL13 ELISA

Human CXCL13 ELISA kit was purchased from R&D Systems (Minneapolis, MN) and performed according to manufacturer specifications and as previously described^23^. Data are reported as concentrations of CXCL13 in peripheral blood sera collected from immunized rhesus macaques, as determined by interpolation using a standard curve.

### B cell sorting and phenotyping

Week 98 cryopreserved PBMC samples were thawed, counted, and CD3 depleted using anti-CD3 NHP microbeads (Miltenyi Biotech, Gaithersburg, MD). Cells were then washed with DPBS and stained with a viability dye (LIVE/DEAD Fixable Aqua Dead Cell Stain, Invitrogen) for 20 min, washed twice in buffer (PBS + 1%FBS), and then surface stained for 30 min at room temperature using commercially available antibodies and 1086cK160Nd11 gp120 hooks conjugated to either AF647 or BV421^24–26^. Cells were then washed and resuspended in 0.04% BSA in PBS and immediately acquired on a upgraded FACS Aria II. HIV specific B cells were identified by sorting on Live Cells, CD14/CD19/CD3 negative cells, CD20 Positive cells, then gp120-BV421, gp120-AF647 dual positive cells. Fluorescently conjugated Abs used for cell surface staining were: IgM BV605 (BD Biosciences cat 562977, Clone G20-127 volume 0.62 μL per 100), CD3 BV650 (BD Biosciences cat 563916, clone SP34-2 volume 1.25 μL 100), CD16 BV650 (BD Biosciences cat 563692, clone 3G8 volume 1.25 μL per 100), CD14 BV650 (BD Biosciences cat 563419, clone M5E2 volume 1.25 μL per 100), CD27 BV711 (Biolegend cat 302834, San Diego, CA, clone 0323 volume 0.62 μL per 100), CD20 BV785 (Biolegend cat 302356, clone 2H7 volume 0.62 μL per 100), CD21 Pe-Cy7 (BD Biosciences cat 561374, clone B-ly4 volume 0.62 μL per 100), and IgG APC-H7 (BD Biosciences cat 561297, clone B56 volume 1.25 μL per 100).

### Antigen-specific B cell single-cell RNA sequencing (scRNA-seq)

Week 98 HIV Env-specific B cells present in peripheral blood were flow-sorted as described above, and gene expression and B cell receptor (BCR) sequencing was performed using scRNA-seq with the 10x Genomics Platform. Briefly, sorted cells were resuspended in PBS + 0.04% BSA. To improve efficiency of single-cell partitioning and minimize cell loss we added 5000 irrelevant cells (murine EL4 cell line, ATCC TIB-39) to each cell suspension. Transcripts from these filler cells are easily removed during initial sequencing data quality control analysis due to species and cell-type mismatch. Single cell suspensions were loaded onto a GemCode Single-Cell instrument (10× Genomics, Pleasanton, CA) and single-cell RNA-seq libraries were then prepared from the single-cell bead emulsions using a 5’ GEX library and VDJ library (10x Genomics) followed by sequencing using 150 cycle high output NextSeq (Illumina, San Diego, CA). Mean reads per cell exceeded 20 thousand for GEX and 5 thousand for VDJ.

### scRNA-seq mapping and analysis

Transcriptome and immune profiling analysis were performed using the Cell Ranger Single Cell Software Suite (version 4.0.0, 10x Genomics) as previously described^63^. Briefly, for transcriptomic analysis, samples were demultiplexed, assembled, filtered, and aligned to the Mmul10 reference (accession NCBI:GCA_003339765.3) followed by unique molecular identifier (UMI) counting. For the immune profiling analysis, samples were demultiplexed, assembled, filtered, and aligned to a custom rhesus macaque VDJ reference. The custom VDJ reference was created by compiling three reference libraries: the default macaque Ig gene library from the software Cloanalyst (https://www.bu.edu/computationalimmunology/research/software/), macaque reference Ig gene segments from IMGT, and macaque reference Ig gene segments from macaque genome sequencing^64^ and formatted for compatibility with Cell Ranger. A text file specifying the sequences of the rhesus macaque inner enrichment primers was also used as described in the documentation for running customized libraries in Cell Ranger that is provided by 10X Genomics. Cell ranger sequences and chain annotations were then used as input into the Cloanalyst software package. Immunogenetics information of rhesus macaque antibody sequences were assigned using the default rhesus macaque Cloanalyst library and sequences were grouped into clones with Cloanalyst. Graph-based cell clustering, dimensionality reduction, and data visualization were performed using the Seurat R package (version 4.0.0). Cells that exhibited >5% mitochondrial transcripts, expressed less than 200 unique transcripts, or more than 2500 unique transcripts were excluded. Additionally, only cells with a single copy of functional light and heavy chain (1:1 functional) in the BCR sequencing are included in the transcriptomic analysis. After applying the above QC criteria we identified a total of 6130 cells (3205 cells from Env immunized, and 2925 cells from Env–HB immunized macaques) to be included in the analysis. Differentially expressed transcripts were determined using the Likelihood-ratio test for single-cell gene expression statistical tests in the Seurat R package. Graphics were generated using Seurat and ggplot2 R packages.

### Binding antigen multiplex assay (BAMA)

Custom BAMA was performed with heat-inactivated plasma samples as previously described^65^. HIV Env-specific antigens were conjugated to polystyrene beads (Bio-Rad Laboratories, Inc., Hercules, CA), and the coupled beads were used to approximate the binding responses of the vaccine-induced antibodies to a panel of envelope glycoproteins. The following panel of antigens was tested: gp70 B case A2 V1V2, 1086.C V1V2, gp70 ConC_V3tags, Con6 gp120/B (106), and 1086.C K160N gp120. The following Env peptides were used: Biotin (Bio) V2.1086 C (Bio-KKKTELKDKKHKVHALFYKLDVVP), Bio V3.C (Bio-KKKNNTRKSIRIGPGQTFYATGDIIGDIRQAHC), Bio V2.B (Bio-KKKTSIRDKVQKEYALFYKLDVVP), C1 Biotin (Bio-KKKMQEDVISLWDQSLKPCVKLTPLCV), and Bio_RV144_ C5.2.C (Bio-KKKSELYKYKVVEIKPLGIAPTKAKRRVVEREKRAV). RIVIG (rhesus immunodeficiency virus immune globulin) was used as positive control. The antigen-conjugated beads were incubated with plasma at a 1:100 dilution. IgG binding in plasma was detected with PE-conjugated mouse anti-monkey IgG antibody (Southern Biotech, Birmingham, AL) at 4 μg/ml. Assays were read on a Bio-Plex 200 instrument (Bio-Rad Laboratories), and binding results were expressed in MFI units. The MFI of beads in wells that did not contain sample (blank wells) was subtracted from sample-specific MFIs within respective assay plates and the blank beads or beads coated with MulV gp70 His6 were used for background. An HIV Env-specific antibody response was considered positive if it had MFI values above the lower detection limit of 100 MFI after background correction. To ensure consistency between assays, 50% effective concentration and maximum MFI values of the positive controls were tracked by Levey-Jennings charts.

### ADCC GTL assay

The ADCC-GTL assay was performed as previously described^66^. Briefly, gp120 coated CEM.NKR_CCR5_ cells (NIH Reagent Program, from A. Trokla) were labeled with TFL4 and the viability marker NFL1 (both from OncoImmunin, Gaithersburg, MD, each at final dilution of 1:1000). After counting and washing, 5000 target cells per well were added to 96-well V-bottom plates and incubated with the Granzyme B (GzB) substrate (OncoImmunin) and Human PBMC effector cells (30:1 effector to target ratio) for 5 min at room temperature. Serial dilutions of plasma samples were then added and the plate was incubated an additional 15 min at room temperature, centrifuged for 1 min at 300 x g, then incubated for 1 h at 37 °C 5% CO_2_. Well contents were then washed and re-suspended in 150 µl 1%FBS in PBS and acquired directly with BD Fortessa flow cytometer (BD Biosciences, San Jose, CA) within 4 h using the High Throughput Sampler (HTS, BD Biosciences). Prior to acquisition with FACSDiva software (BD Biosciences), the area scaling factor was adjusted to ensure that cell singlets (CEM.NKR_CCR5_ CD4^+^ T cells) indicated equivalent ratios of FSC-H and FSC-A. Flow cytometry data analysis was performed using FlowJo 10.8.0 software (BD Biosciences).

### Infected cell antibody binding assay (ICABA)

Indirect surface staining was used to measure the ability of plasma antibodies to bind to the surface of HIV-1 infected cells using methods similar to those previously described^28,65^. Briefly, CEM.NKR_CCR5_ cells were mock infected or infected with an HIV-1 infectious molecular clone virus expressing the C.1086, C.TV-1, or AE.CM235 Env protein. The cells were then washed and incubated with a 1:100 dilution of plasma samples for 2 h at 37 °C and then stained with Live/Dead Aqua Dead Cell Stain (Thermo Fisher Scientific) to exclude dead cells from analysis. Cells were next washed and then permeabilized with Cytofix/Cytoperm solution (BD Biosciences) prior to staining with RD1-conjugated anti-p24 antibody KC57 (Beckman Coulter, Inc., Indianapolis, IN) and fluorescein isothiocyanate (FITC)-conjugated goat anti-rhesus IgG (H + L) polyclonal antiserum (Southern Biotech). Cells positive for serum antibody binding were defined as viable, p24 positive, and FITC positive. Final results were reported as the change in FITC median fluorescent intensity (MFI) for post-vaccination samples compared to the pre-vaccination sample; gated on the live, infected cell population (p24 positive cells) after subtraction of the MFI observed for staining of cells stained with secondary antibody alone and mock-infected cells.

### ADCC-Luc

The ADCC-Luc assay was performed as previously described^57,67^. Briefly, CEM.NKR_CCR5_ were infected with replication competent infectious molecular clones (IMCs) of C.1086, C.TV-1, or AE.CM235. These contain C.1086, C.TV-1, or AE.CM235 envelope sequences on NL-LucR.T2A backbone^68^. The infection of the cells was confirmed with p24 intracellular staining. The infected cells were co-plated with the human NK92 cell line engineered to express the 158I variant of rhesus macaque FcγRIIIa^69^ at a 10:1 ratio for 6 h in complete media with serially diluted plasma from the vaccinated animals. At the end of the 6-hour incubation, Viviren (Promega, Madison, WI) was added to the plate and the luminesce of each well was measured. ADCC activity, reported as percent specific killing, was calculated from the change in relative light units (RLU) resulting from the loss of intact target cells in wells containing effector and target cells in the presence of serum samples compared to RLU in control wells containing target cells and effector cells alone, and after baseline subtraction using the pre-vaccination time point Week 60.

### Neutralization

Neutralization was measured as the ability of serum samples to reduce virus infection of TZM-bl cells as previously described^70^. Serum was incubated with subtype B tier 1 isolate (B.MN.3), tier 2 isolate C.TV-1, or tier 1 isolate AE.TH023.6 pseudoviruses for 45 min at 37 °C (29, 30), plated with TZM-bl cells, and then allowed to incubate for 48 h. A luciferase reagent (Bright-Glo; Promega) was added, and luminescence was measured. Results were reported as the 50% inhibitory dilution (ID50), which is the dilution of serum resulting in 50% reduction in luminescence compared to virus control wells.

### Statistical analysis

Spearman’s rank correlation coefficient was used to calculate correlations between immunologic markers. Mann-Whitney *U* tests were used to compare these markers between Env and Env-HB groups and the resulting *p*-values were adjusted for multiple testing to control for the false discovery rate (FDR) with the Benjamini-Hockberg method. Random forest models were developed as a classification method and to identify the immunologic markers that classified each vaccine group. The variable importance measures for each random forest model were calculated using the mean decrease in Gini impurity as a ranking measure which determines how the model’s accuracy decreased when a variable was removed. The out-of-bag estimate error rate was used to assess the accuracy of each random forest. We also used lasso-regularized logistic regression models as an alternative approach to identify the immunologic variables that classified the two vaccine groups, and compared these results to random forest results. The penalty parameter λ was selected at the value with the lowest binomial deviance after 10-fold leave-one-out cross-validation. The penalized model was then fit using the minimum λ value and the non-zero coefficients were extracted.

### Immunoblotting

Monolayers of DF-1 cells (ATCC #CRL-3586) were mock-infected or infected with MVA_Env, MVA_Env-HBsAg, or parental MVA at a multiplicity of infection of 3 for 24 h. Cells were then washed with PBS and lysed by repeat freeze-thaw cycles, sonication, and scraping in the presence of protease inhibitor (HALT, Protease Inhibitor Cocktail, Thermo-Fisher Scientific). The total protein concentration of the lysates was determined using the Bio-Rad Quick Start^TM^ Bradford assay (Bio-Rad Laboratories, Inc). Equal amounts of total protein (2 μg each) and 5 μL protein standards solution (Precision Plus Unstained Protein Standards, Bio-Rad Laboratories, Inc.) were loaded onto 4% to 20% gradient polyacrylamide gels (Mini-PROTEAN® TGX, Bio-Rad Laboratories) in Laemmli buffer with β-mercaptoethanol and separated by electrophoresis on the BioRad Mini-PROTEAN Tetra System. The proteins were transferred to PVDF membranes using the TransBlot® Turbo System (Bio-Rad Laboratories, Inc.) and blocked for 1 h in 2% BSA in tris-buffered saline (TBS) with 0.05% tween (TBST). Primary antibodies (anti-Env, mAb 2G12, DHVI Protein Production Facility; and anti-HBsAg, mAb ab68520, Abcam) were diluted in 1% BSA TBST and incubated with the membrane for 1 h at 4 °C. The membranes were then washed extensively in TBS. Secondary detection reagents (anti-human HRP for anti-Env immunoblot, Sigma Chemical; and Streptactin HRP for anti-HBsAg immunoblot, Bio-Rad Laboratories) were diluted in 1% BSA TBST and incubated with the membranes for 1 h at 4 °C, followed by extensive washing with TBST and one wash with TBS. Band visualization was performed using the Clarity^TM^ Western ECL substrate (Bio-Rad Laboratories, Inc) according to manufactures’ instructions, and images were acquired on the Bio-Rad ChemiDoc imaging system. All blots were derived from the same experiment and were processed in parallel.

### Negative stain electron microscopy (NSEM)

NSEM analysis was performed via a Core Service of the Duke Human Vaccine Institute under the direction of Dr. R.J. Edwards. A frozen aliquot of the Env–HB conjugate vaccine from −80 °C was thawed at room temperature in an aluminum block for 5 min. Sample was then diluted to 40 µg/ml with 0.02 g/dl Ruthenium Red in HBS (20 mM HEPES, 150 mM NaCl pH 7.4) buffer. After 10–15 min incubation, the sample was applied to a glow-discharged carbon-coated EM grid for 8–10 s, blotted, consecutively rinsed with 2 drops of 1/20X HBS, and stained with 2 g/dL uranyl formate for 1 min, blotted and air-dried. Grids were examined on a Philips EM420 electron microscope operating at 120 kV and nominal magnification of 49,000x, and 10 images were collected on a 76 Mpix CCD camera at 2.4 Å/pixel.

### Anti-HIV-1 Envelope Enzyme-Linked Immunosorbent Assay (ELISA)

Anti-HIV Env ELISA was performed using the same general approach as described for anti-HBsAg ELISA. Briefly, high-binding plates were coated with the Env protein vaccine or the Env-HB protein conjugate vaccine at 2 μg/mL overnight at 4 °C using Coating Buffer provided in the ThemoFisher ELISA Buffer Kit (ThermoFisher Scientific). Plates were then washed in Wash Buffer and blocked for 1 h using Assay Buffer provided in the kit. HIV-1 specific human monoclonal antibodies targeting different regions of the HIV-1 Env (mAbs VRC01, 2G12, A32, CH59, 830 A, PG9, Ab3074, 10-1074) or the anti-influenza control mAb CH65 were serially diluted (6 serial 5-fold dilutions starting at 0.5 μg/mL) in Assay Buffer and added to the plates for 1 h at room temperature. All conditions were tested in duplicate wells. Plates were washed and binding of primary antibodies was detected by incubation with goat anti-Human IgG HRP (Jackson ImmunoResearch, Inc., West Grove, PA), diluted in Assay Buffer, for 1 h at room temperature. Plates were developed using the buffers and chromogen solution provided in the kit according to manufacturer’s recommendations. Plates were read on a Perkin-Elmer VICTOR® Nivo^TM^ reader at 450 nm within 30 min.

### Reporting summary

Further information on research design is available in the Nature Research Reporting Summary linked to this article.

### Supplementary information

Supplementary Info

REPORTING SUMMARY

## Data Availability

All data generated for this work is available in a Gitlab repository.

## Code availability

All code used for analysis are available in a Gitlab repository.
