# Supplementary material for: Conjugation of HIV-1 envelope to hepatitis B surface antigen alters vaccine responses in rhesus macaques
Source: NPJ Vaccines. 2023 Nov 24;8:183. doi: 10.1038/s41541-023-00775-y (PMC10673864; doi:10.1038/s41541-023-00775-y)
Supplement: Supplementary file 1 — Supplementary Info [file 41541_2023_775_MOESM1_ESM.pdf]

SUPPLEMENTARY MATERIAL

Manuscript: Vaccination with a HIV-1 envelope and hepatitis B surface antigen conjugate vaccine alters vaccine-elicited responses in juvenile rhesus macaques

Authors: Nettere et al.

Supplementary Data

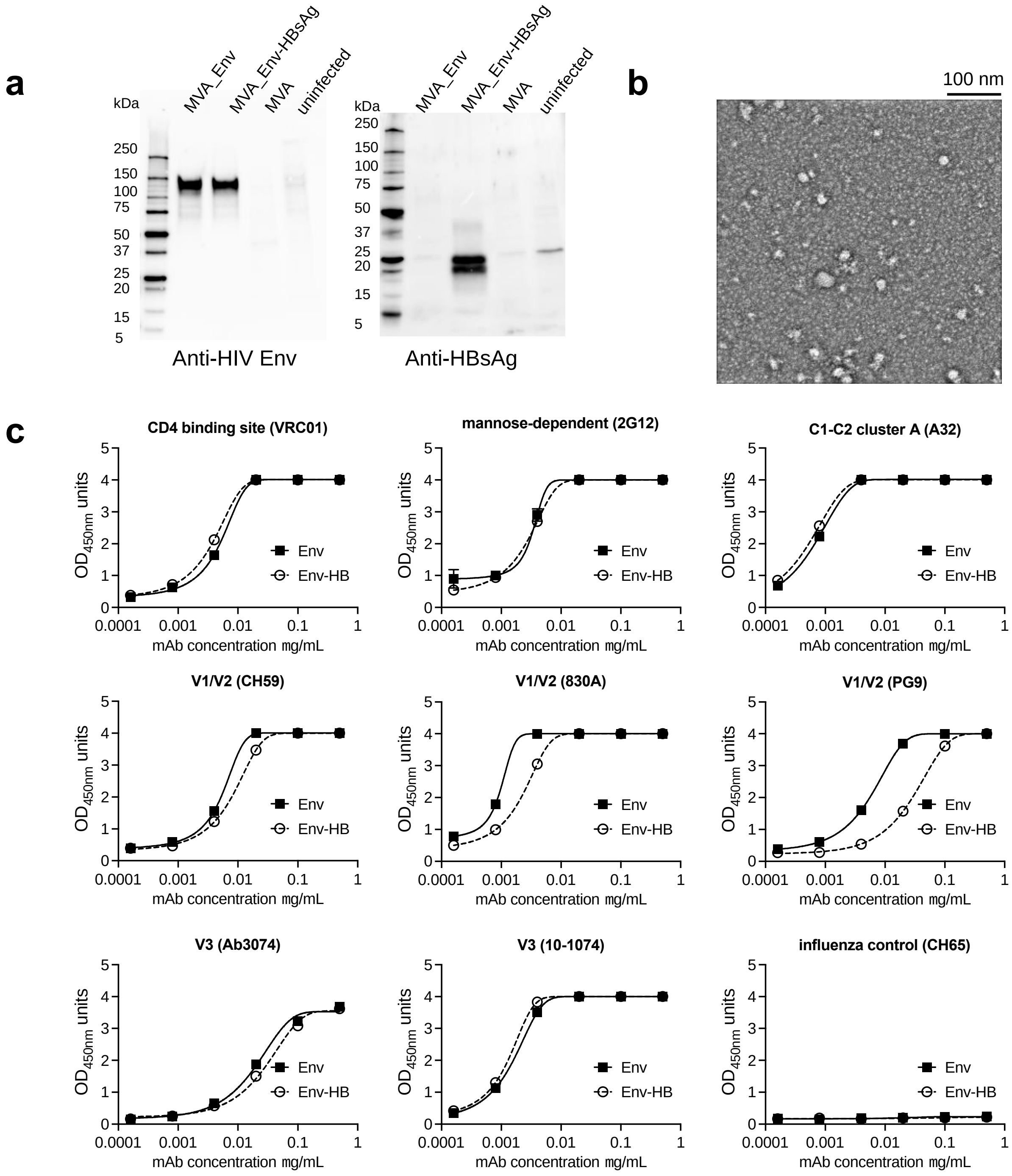

Supplementary Figure 1. Characterization of vaccine components. a. Detection of HIV-1 Env and HBsAg in lysates of cells infected with MVA_Env and MVA_Env-HBsAg by immunoblotting. Cell lysates were collected, and immunoblotting was performed on equal amounts of total protein using anti-Env (mAb 2G12, left panel) and anti-HBsAg (mAb ab68520, right panel) primary detection antibodies. As shown, cells infected with both the MVA_Env and MVA_Env-HBsAg viruses produced HIV Env, but only cells infected with MVA_Env-HBsAg virus also produced HBsAg. Neither protein was produced by cells infected with parental MVA. **b.** Negative stain electron micrograph of the Env-HB protein conjugate indicated a “lawn” of small particles consistent with the expected size of conjugate monomers with a minor fraction of larger protein aggregates. Scale bar represents 100 nm **c.** Binding of a panel of

HIV-specific monoclonal antibodies to the Env and ENV-HB conjugate protein vaccines as determined by ELISA. Anti-influenza antibody CH65 was included as a negative control.
