# Supplementary material for: Conjugation of HIV-1 envelope to hepatitis B surface antigen alters vaccine responses in rhesus macaques
Source: NPJ Vaccines. 2023 Nov 24;8:183. doi: 10.1038/s41541-023-00775-y (PMC10673864; doi:10.1038/s41541-023-00775-y)
Supplement: Supplementary file 2 — REPORTING SUMMARY [file 41541_2023_775_MOESM2_ESM.pdf]

nature portfolio

Corresponding author(s): Justin Pollara

Last updated by author(s): Jul 24, 2023

Reporting Summary

Nature Portfolio wishes to improve the reproducibility of the work that we publish. This form provides structure for consistency and transparency in reporting. For further information on Nature Portfolio policies, see our Editorial Policies and the Editorial Policy Checklist.

Statistics

For all statistical analyses, confirm that the following items are present in the figure legend, table legend, main text, or Methods section.

n/a	Confirmed	
☐	☒	The exact sample size (*n*) for each experimental group/condition, given as a discrete number and unit of measurement
☐	☒	A statement on whether measurements were taken from distinct samples or whether the same sample was measured repeatedly
☐	☒	The statistical test(s) used AND whether they are one- or two-sided<br>*Only common tests should be described solely by name; describe more complex techniques in the Methods section.*
☒	☐	A description of all covariates tested
☐	☒	A description of any assumptions or corrections, such as tests of normality and adjustment for multiple comparisons
☐	☒	A full description of the statistical parameters including central tendency (e.g. means) or other basic estimates (e.g. regression coefficient) AND variation (e.g. standard deviation) or associated estimates of uncertainty (e.g. confidence intervals)
☐	☒	For null hypothesis testing, the test statistic (e.g. *F*, *t*, *r*) with confidence intervals, effect sizes, degrees of freedom and *P* value noted<br>*Give P values as exact values whenever suitable.*
☒	☐	For Bayesian analysis, information on the choice of priors and Markov chain Monte Carlo settings
☒	☐	For hierarchical and complex designs, identification of the appropriate level for tests and full reporting of outcomes
☒	☐	Estimates of effect sizes (e.g. Cohen's *d*, Pearson's *r*), indicating how they were calculated

Our web collection on statistics for biologists contains articles on many of the points above.

Software and code

Policy information about availability of computer code

Data collection	no software used
Data analysis	https://gitlab.oit.duke.edu/su57/r25-cfar-2021-pollara

For manuscripts utilizing custom algorithms or software that are central to the research but not yet described in published literature, software must be made available to editors and reviewers. We strongly encourage code deposition in a community repository (e.g. GitHub). See the Nature Portfolio guidelines for submitting code & software for further information.

Data

Policy information about availability of data

All manuscripts must include a data availability statement. This statement should provide the following information, where applicable:

- Accession codes, unique identifiers, or web links for publicly available datasets
- A description of any restrictions on data availability
- For clinical datasets or third party data, please ensure that the statement adheres to our policy

All data generated for this work is available and the code used for analysis are available in a Gitlab repository. https://gitlab.oit.duke.edu/su57/r25-cfar-2021-pollara

Research involving human participants, their data, or biological material

Policy information about studies with human participants or human data. See also policy information about sex, gender (identity/presentation), and sexual orientation and race, ethnicity and racism.

Reporting on sex and gender	n/a
Reporting on race, ethnicity, or other socially relevant groupings	n/a
Population characteristics	n/a
Recruitment	n/a
Ethics oversight	n/a

Note that full information on the approval of the study protocol must also be provided in the manuscript.

Field-specific reporting

Please select the one below that is the best fit for your research. If you are not sure, read the appropriate sections before making your selection.

☒ Life sciences ☐ Behavioural & social sciences ☐ Ecological, evolutionary & environmental sciences

For a reference copy of the document with all sections, see nature.com/documents/nr-reporting-summary-flat.pdf

Life sciences study design

All studies must disclose on these points even when the disclosure is negative.

Sample size	Sample size was chosen based on a primary outcome of interest, rate of somatic hypermutation. With n=5 per group, there is 80% power at the 0.05 significance level to detect a difference of 2 standard deviations between the vaccinated and control group values with a two-sided test.
Data exclusions	no data excluded
Replication	Individual assays were completed with replicates and all data was included.
Randomization	randomization was achieved through assignment at birth by birth order with accounting for balancing of sex
Blinding	investigators executing individual experiments were blinded to treatment group

Reporting for specific materials, systems and methods

We require information from authors about some types of materials, experimental systems and methods used in many studies. Here, indicate whether each material, system or method listed is relevant to your study. If you are not sure if a list item applies to your research, read the appropriate section before selecting a response.

Materials & experimental systems

n/a	Involved in the study	
☐	☒	Antibodies
☐	☒	Eukaryotic cell lines
☒	☐	Palaeontology and archaeology
☐	☒	Animals and other organisms
☒	☐	Clinical data
☒	☐	Dual use research of concern
☒	☐	Plants

Methods

n/a	Involved in the study	
☒	☐	ChIP-seq
☐	☒	Flow cytometry
☒	☐	MRI-based neuroimaging

Antibodies

Antibodies used	PD-1 BV421 (Biolegend, clone EH12.2H7, lot B227208),

CD8a BV570 (Biolegend, clone RPA-T8, lot B333843),

CCR7 BV605 (BD Biosciences, clone 3D12, lot 0209411),

CD25 BV650 (Biolegend, clone BC96, lot B325964),

CD4 BV711 (Biolegend, clone OKT4, lot B311962),

CD45RA BV785 (BD Biosciences, clone 5H9, lot 1049498),

ICOS BB515 (BD Biosciences, clone C398.4A lot 0276637),

CCR6 BB700 (BD Biosciences, clone 11A9, lot 1020712),

OX40 PE (BD Biosciences, clone L106, lot),

CXCR3 PE-CF594 (BD Biosciences, clone 1C6 lot 104031),

CD69 PE-Cy5 (Biolegend, clone FN50, lot B326045),

CXCR5 Biotin (eBioscience, San Diego, CA, clone MU5UBEE,lot 2220914),

CD137 AF647 (Biolegend, clone 4B4-1, lotB308591),

CD3 APC-Cy7 (BD Biosciences, clone SP34-2).

PeCy7-Streptavidin (Biolegend, lot B300682)

IgM BV605 (BD Biosciences, Clone G20-127, lot 0282570),

CD3 BV650 (BD Biosciences, clone SP34-2, lot 0280269),

CD16 BV650 (BD Biosciences, clone 3G8, lot),

CD14 BV650 (BD Biosciences, clone M5E2, lot 8339727),

CD27 BV711 (Biolegend, San Diego, CA, clone M-T271, lot 1032599),

CD20 BV785 (Biolegend, clone 2H7, lot B320041),

CD21 Pe-Cy7 (BD Biosciences, clone B-ly4, lot 0058165), and

IgG APC-H7 (BD Biosciences, clone B56, lot 0255669).

CH65_G1M17 lot 5NSR
2G12_AAA lot 196JCA
VRC01 Amp drug product lot 17-597
A32_G1.4A lot 230203PPf
PG9_4A lot 180614PPF
101074_4A lot 58JKC
AbSZP3074_A1 lot 174AMS
830A lot 5/1/18 TAV
CH59_4A

Validation

Antibodies were purchased from reputable vendors with documented validation and quality assurance, stored according to the manufacturer's recommendations, and not used beyond the expiration date.

The specificity and optimal concentration of all antibodies for use in flow cytometry-based studies was be evaluated by titration using PBMC samples, and validated by comparison to prior data obtained by our laboratory.

Non-commercially available proteins were produced by transfection of 293T cells using plasmids designed based on published sequences, with final plasmid sequences confirmed by vector sequencing. Proteins are purified by affinity chromatography and size exclusion chromatography, and quality assessed by running reduced and non-reduced SDS page gels. All proteins are stored at -80 degrees. New lots of proteins are tested to ensure consistent performance in assays.

Eukaryotic cell lines

Policy information about cell lines and Sex and Gender in Research

Cell line source(s)

CEM.NKR.CCR5 (From the NIH AIDS Reagent Program, Division of AIDS, NIAID, NIH from Dr. Trkola)
NK92 Rh. CD16 (Generation of these cells is described in PMID: 36131913)
DF-1 (From the American Type Culture Collection)

Authentication | All lines were confirmed for expression of key markers by flow cytometery

Mycoplasma contamination | All cell lines tested negative for mycoplasma at time of thaw

Commonly misidentified lines (See ICLAC register) | n/a

Animals and other research organisms

Policy information about studies involving animals; ARRIVE guidelines recommended for reporting animal research, and Sex and Gender in Research

Laboratory animals	Indian Rhesus macaque, (Mucaca mulatta) ages from birth to 120 weeks of age included in study
Wild animals	n/a
Reporting on sex	vaccine arms were balanced for sex and single cell RNA seq analysis evaluated sex differences between animals and found none (figure 4)
Field-collected samples	n/a
Ethics oversight	IACUC of the university of California, Davis

Note that full information on the approval of the study protocol must also be provided in the manuscript.

Flow Cytometry

Plots

Confirm that:

☒ The axis labels state the marker and fluorochrome used (e.g. CD4-FITC).

☒ The axis scales are clearly visible. Include numbers along axes only for bottom left plot of group (a 'group' is an analysis of identical markers).

☒ All plots are contour plots with outliers or pseudocolor plots.

☒ A numerical value for number of cells or percentage (with statistics) is provided.

Methodology

Sample preparation	Detailed sample preparation can be found in methods. Briefly, blood products were obtained from venipunture. Plasma was separated by centrifugation and stored at -80 deg C, PBMCs were processed using Ficoll separation and stored in liquid nitrogen. Lymph node biopsy samples were processed via mechanical separation and also stored in liquid nitrogen.
Instrument	Figure 2 data: LSR Fortessa, Figure 3 data: Aria IIU
Software	Analyzed on FlowJo version 10.8.0
Cell population abundance	Cells were not recounted post sort to minimize cell loss. 10X genomics used alignment to exclude transcriptional changes from non-B cells.
Gating strategy	Gating strategy is provided in the main figures for TFH data and B cell phenotyping

☒ Tick this box to confirm that a figure exemplifying the gating strategy is provided in the Supplementary Information.
